# Study on the mechanism of Baihe Dihuang decoction in treating menopausal syndrome based on network pharmacology

**DOI:** 10.1097/MD.0000000000033189

**Published:** 2023-05-17

**Authors:** Mingmin Tian, Anming Yang, Qinwei Lu, Xin Zhang, Guangjie Liu, Gaofeng Liu

**Affiliations:** a School of Chinese Medicine, Jiangxi University of Traditional Chinese Medicine, Nanchang, P. R. China; b Department of Neurosurgery, Nanfang Hospital, Southern Medical University, Guangzhou, P. R. China; c School of Integrated Chinese and Western Medicine, Nanjing University of Chinese Medicine, Nanjing, P. R. China; d School of Traditional Chinese Medicine, Southern Medical University, Guangzhou, P. R. China.

**Keywords:** analysis of enrichment, Baihe Dihuang decoction, mechanism of action, menopausal syndrome, network pharmacology, targets

## Abstract

Menopausal syndrome (MS) refers to a series of symptoms with autonomic nervous system dysfunction caused by decreased sex hormones before and after menopause. Baihe Dihuang (BHDH) decoction positively affects MS, but its mechanism remains unclear. This study aimed to reveal the underlying mechanism through network pharmacology. The components of the BHDH Decoction were found through HERB, while corresponding targets were obtained from the HERB, Drug Bank, NPASS, Targetnet, and Swisstarget databases. The MS targets were obtained from GeneCards and OMIM. STRING was used to construct the protein-protein interaction networks. OmicShare tools were used for Gene Ontology and Kyoto encyclopedia of genes and genomes analyses. Finally, Autodock Vina 1.1.2 software (https://vina.scripps.edu/downloads/) was used for molecular alignment to verify whether the main active ingredients and key targets had good binding activity. We screened out 27 active ingredients and 251 effective targets of BHDH Decoction, 3405 MS-related targets, and 133 intersection targets between BHDH Decoction and MS. Protein-protein interaction network identified tumor protein P53, Serine/threonine-protein kinase AKT, epidermal growth factor receptor, Estrogen Receptor 1, and jun proto-oncogene as critical targets. Gene ontology analysis showed that these targets were mainly involved in the cellular response to chemical stimulus, response to oxygen-containing compound, cellular response to endogenous stimulus, response to an organic substance, and response to chemical, etc. Kyoto encyclopedia of genes and genomes pathways were mainly enriched in endocrine resistance, pathways in cancer, and the ErbB signaling pathway, etc. Molecular docking results showed that emodin and stigmasterol are strongly associated with Serine/threonine-protein kinase AKT, Estrogen Receptor 1, epidermal growth factor receptor, sarcoma gene, and tumor protein P53. This study preliminarily revealed the multi-component, multi-target, and multi-channel mechanism of BHDH Decoction in treating MS. It provides a reference for in vitro and in vivo research and clinical application of BHDH Decoction in the treatment of MS.

## 1. Introduction

Menopausal syndrome (MS), also known as perimenopausal syndrome, is characterized by autonomic nervous system dysfunction caused by ovarian failure, pituitary hyperfunction, or low estrogen level, often accompanied by symptoms such as menstrual disorder, hot flashes, hyperhidrosis, sleep disorder, and mood disorder.^[1]^ Western medicine treats perimenopausal syndrome with menopausal hormone therapy and psychotherapy as the primary means. Supplementing with exogenous estrogen to maintain the function and state of estrogen target organs in vivo has a definite curative effect on alleviating related clinical symptoms.^[2]^ However, studies have shown that long-term estrogen use may increase the risk of breast cancer, endometrial cancer, and cardiovascular diseases.^[3]^

MS belongs to the categories of “dirty mania,” “depression syndrome,” and “lily disease” in traditional Chinese medicine. Its onset is caused by the gradual decline of kidney qi, exhaustion of “tiangui,” and imbalance of yin and yang.^[4]^ The treatment of Menopause syndrome in traditional Chinese medicine mainly focuses on nourishing the kidneys and yin, calming the heart, and tranquilizing the mind. Baihe Dihuang (BHDH) decoction originated from the “Synopsis of Golden Chamber” in the Han Dynasty, which is a particular prescription for the treatment of “lily disease.” In this prescription, Lily is used as the principal drug, which has the effects of nourishing yin, moistening lungs, clearing the heart, and tranquilizing the mind; Rehmanniae Radix is used as an assistant, which has the function of clearing heat and cooling blood, nourishing yin, and promoting fluid. The compatibility of the lily bulb and Rehmanniae Radix has the functions of Nourishing Yin, clearing heat, and tonifying the heart and lungs. Modern research shows that BHDH Decoction has antidepression and antianxiety functions, regulates subhealth, and improves sleep. This is especially true in treating MS, which causes hot flashes, sweating, sleep disorders, and emotional disorders.^[5]^

Some studies have explored the mechanism of BHDH Decoction in treating MS through clinical efficacy or animal experiments.^[6,7]^ However, because of the complexity of the active components in BHDH Decoction and the diversity of potential regulatory targets in the human body, it is challenging to describe the scientific basis and potential pharmacological mechanism of BHDH Decoction in the treatment of MS using conventional methods.

This study used network pharmacology to explore the potential mechanism of BHDH in the treatment of MS. Molecular docking technology was used to virtually combine the practical active components and receptor protein molecules in BHDH Decoction to explore its action targets and binding sites and elaborate the synergistic mechanism of multi-component, multi-target, and multi-channel BHDH Decoction, to provide a theoretical basis for the clinical treatment of MS.

## 2. Materials and methods

### 2.1. Schematic diagram

**Figure 1.**
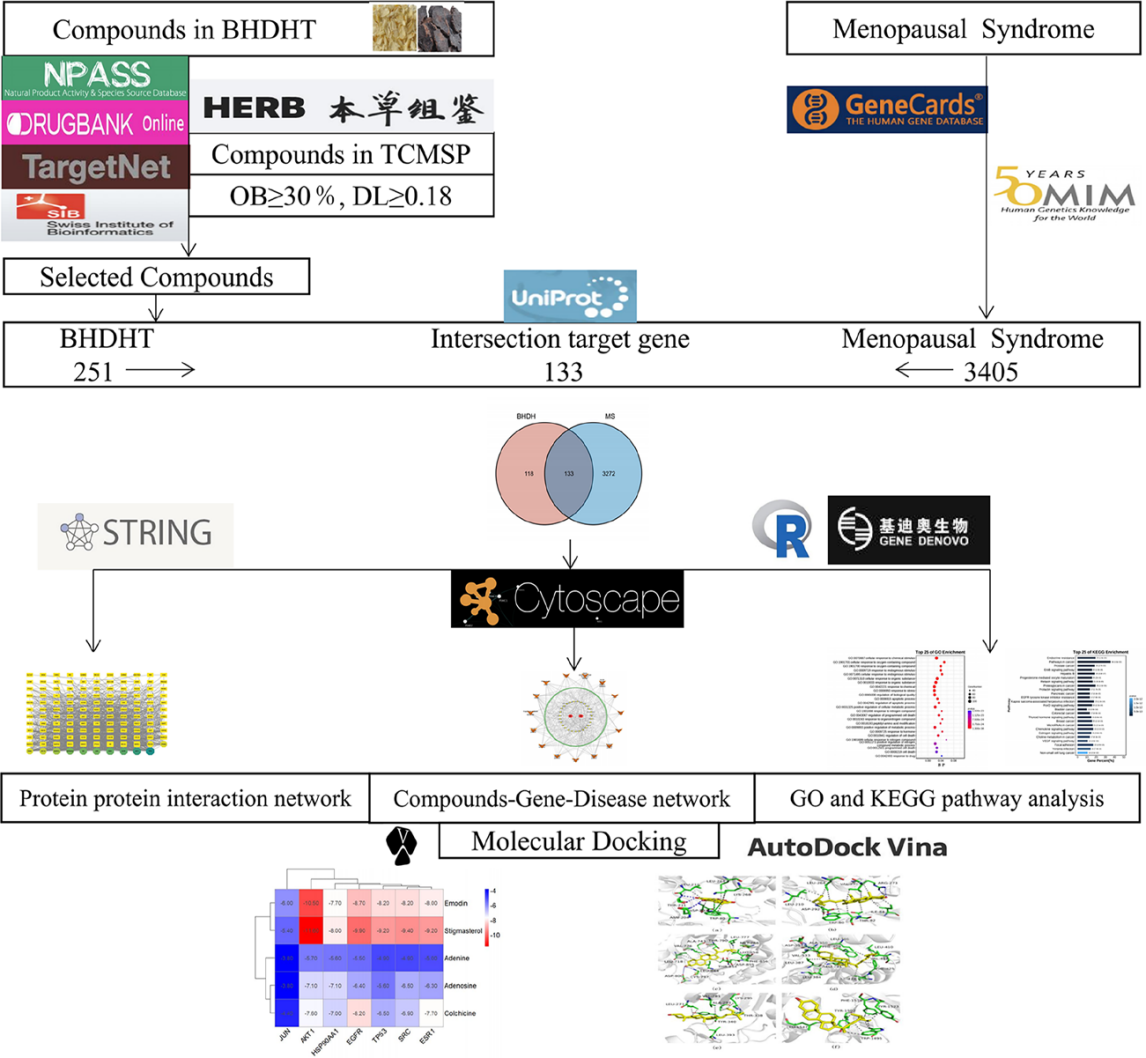
Flow chart of research methods.

Based on network pharmacology and molecular docking methods, we studied the mechanism of BHDH Decoction in treating MS, and the flow chart of the research methods is shown in Figure 1.

### 2.2. Compound collection and target prediction of Baihe Dihuang decoction

HERB (http://herb.ac.cn/) is a high-throughput and reference-guided database of traditional Chinese medicine. Its Chinese name is “Ben Cao Zu Jian,” which integrates multiple databases of traditional Chinese medicine and contains the most comprehensive list of traditional Chinese medicines and components.^[8]^

By searching the words “Lily” and “Rehmanniae Radix,” we obtained the compounds of each herb in BHDH from HERB. The experimentally verified targets of these compounds were downloaded from the Drug Bank (https://go.drugbank.com/) and the NPASS (http://bidd.group/NPASS/) databases. All components of BHDH were selected based on oral bioavailability (OB) ≥ 30% and drug-like (DL) ≥ 0.18 from the pharmacological database and analysis platform of the Chinese medicine system (TCMSP, https://old.tcmsp-e.com/tcmsp.php). The bioactive compounds that contributed to the therapeutic effect of BHDH were selected, and those with poor pharmacological properties and drug potency were removed. The targets of the 3 databases were intersected, and the intersected targets were combined with the corresponding targets obtained from the Drug Bank and NPASS databases, which are the final targets of BHDH. UniProt (https://www.uniprot.org/) was used to standardize the targets.

### 2.3. Prediction target of menopausal syndrome

Referring to the research method of Lin et al,^[9]^ combined with our study, we obtained MS-related targets from the following 2 databases. GeneCards (https://www.genecards.org/) is a searchable, integrative database that provides comprehensive, user-friendly information on all annotated and predicted human genes. The knowledgebase automatically integrates gene-centric data from 150 web sources, including genomic, transcriptomic, proteomic, genetic, clinical, and functional information; Online Mendelian Inheritance in Man (OMIM, https://mirror.omim.org) is a comprehensive and authoritative database for studying the relationship between human phenotype and genotype, which contains all known Mendelian diseases and information of more than 16,000 genes (covering more than half of the known genes of human beings). OMIM does not create these data but is a systematic collation and integration of published research results, which were updated daily and obtained free of charge. We use “Menopausal syndrome” as the keyword to screen disease targets in each database and summarize the obtained targets to remove the repeated values. Finally, the obtained targets were standardized using UniProt (https://www.uniprot.org/).

### 2.4. Construction and analysis of the drug-component-target network

A Venn diagram was drawn using R language to obtain the target of drug-disease interaction, namely the target of the BHDH Decoction for MS. We used Cytoscape (https://cytoscape.org/, version 3.8.0) to construct the drug-component-target network diagram. Cytoscape is an open-source software platform for visualizing molecular interaction networks and biological pathways and integrating these networks with annotations, gene expression profiles, and other state data. The software was used to analyze the network parameters, including degree, betweenness centrality (BC), and closeness centrality (CC). We used this software to screen the core components and targets of the BHDHT Decoction and the relationship between them.

### 2.5. Protein-protein interaction (PPI) network construction and module screening

STRING (https://string-db.org/) is a database of known and predicted PPI. Interactions include direct (physical) and indirect (functional) associations; they originate from computational prediction, knowledge transfer between organisms, and interactions aggregated from other (primary) databases. We imported the interaction target into STRING to identify the PPI information and visualize the network using Cytoscape. In addition, through further analysis of the PPI network using the MCODE plug-in, potentially important protein function modules were obtained to analyze and explain the biological process in which they participate.

### 2.6. Gene ontology (GO) and Kyoto encyclopedia of genes and genomes (KEGG) enrichment analysis

GO and KEGG enrichment analyses were performed using the OmicShare tools (www.omicshare.com/tools), a free online platform for data analysis, and *P* ≤ .01 was considered statistically significant.

### 2.7. Molecular docking

To verify whether the compound had an excellent binding activity with the target, we selected the top 5 hub components of the drug-composition-target network and the top 7 hub targets of the drug-disease target PPI network for molecular docking. We downloaded the 3D structure of the compound from PubChem (https://pubchem.ncbi.nlm.nih.gov/) and saved it in SDF format, then imported it into ChemDraw 3D, used the MM2 module to minimize the energy, obtained the advantage idea of the lowest energy, and saved it in the MOL2 file. The protein structure was downloaded from the Protein Data Bank (PDB, https://www.rcsb.org/) and visualized using PYMOL. After dehydration, hydrogenation, charge calculation, and a combination of nonpolar hydrogen with MGtools 1.5.6, the ligand and receptor were stored as PDPQT. Autodock vina 1.1.2 was used to dock the ligand with the receptor, and ideas with high scores were visualized using PYMOL and Discovery Studio.

## 3. Result

### 3.1. Identification of active compounds and target prediction of menopausal syndrome

A total of 156 compounds from the 2 herbs in the BHDH Decoction were obtained from the HERB database. After screening and removing the repeat values, we obtained 27 active compounds, including 14 from Lily and 13 from Rehmanniae Radix. Finally, 251 targets were obtained for the BHDH Decoction (See Form 1 and 2 for details, Supplemental Digital Content, http://links.lww.com/MD/J25; http://links.lww.com/MD/J26).

### 3.2. Prediction target of menopausal syndrome

The number of targets associated with MS obtained from the Genecards and OMIM databases was 2816 and 645, respectively. We summarized the obtained goals and removed duplicates, resulting in 3405 MS-related targets.

### 3.3. Construction and analysis of the drug-component-target network

**Figure 2.**
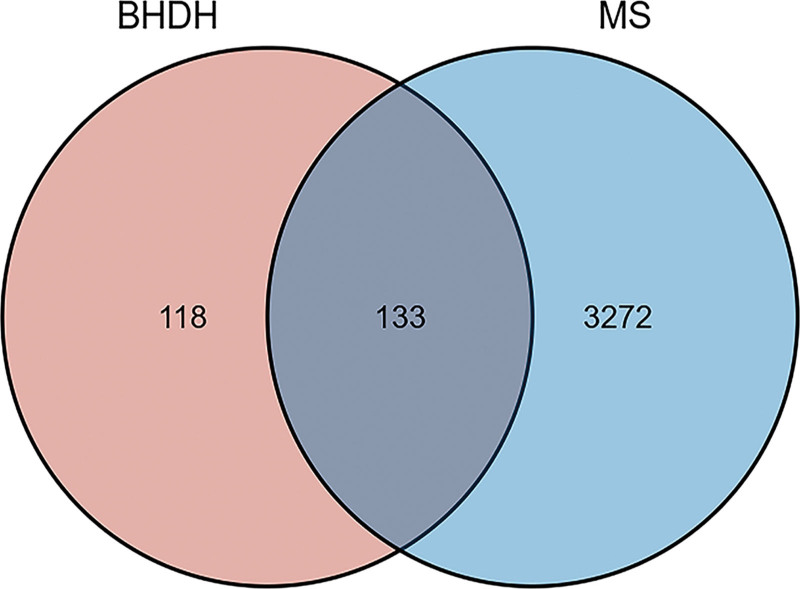
Venn diagram of MS-related targets and targets of BHDH Decoction. MS = menopausal syndrome.

**Figure 3.**
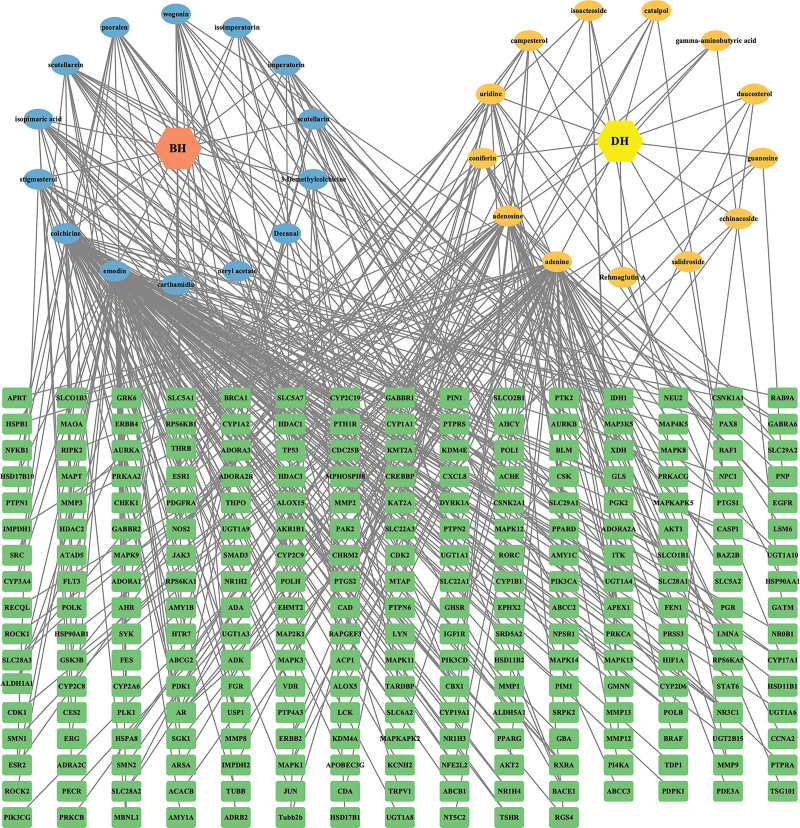
Drug-components-targets network (The circular nodes represent components, green boxes represent drug-disease targets, and the diamond node represents BHDH Decoction).

We used the R language to analyze the identified targets and climacteric syndrome-related targets of the BHDH Decoction (as shown in the Venn diagram, Fig. 2) and obtained 133 drug-disease targets. According to the selected drug-disease targets and their matching relationship with active ingredients, 27 active ingredients that could be targeted to treat MS were identified among 156 active ingredients (Table 1). By inputting the relationship between these active ingredients and drug-disease targets into Cytoscape software, we got a drug-ingredient-target network with 280 nodes and 436 edges (Fig. 3). Cytoscape network analyzer analysis tool was used to analyze the network characteristic parameters and obtain the BC, CC, and degree of each component. The results predicted that emodin (BC = 0.465754816, CC = 0.433903577, degree = 97) would be the hub component of BHDH Decoction in the treatment of menopausal syndrome, followed by adenine (BC = 0.306427916, CC = 0.369536424, degree = 61), colchicine (BC = 0.194159545, CC = 0.380627558, degree = 52), and adenosine (BC = 0.17294926, CC = 0.356321839, degree = 35).

**Table 1:** The main active ingredients of BHDH Decoction.

NO.	Ingredient id	Molecule name	Source	Betweenness Centrality	Closeness Centrality	Degree
1	HBIN025041	Emodin	Bulbus Lilii	0.465754816	0.433903577	97
2	HBIN014684	Adenine	Radix Rehmanniae	0.306427916	0.369536424	61
3	HBIN021260	Colchicine	Bulbus Lilii	0.194159545	0.380627558	52
4	HBIN014693	Adenosine	Radix Rehmanniae	0.17294926	0.356321839	35
5	HBIN044918	Stigmasterol	Bulbus Lilii	0.055647493	0.345724907	19
6	HBIN031031	Isopimaric acid	Bulbus Lilii	0.071655433	0.327080891	18
7	HBIN043527	Scutellarein	Bulbus Lilii	0.087924505	0.306256861	17
8	HBIN041121	Psoralen	Bulbus Lilii	0.033490883	0.287332647	14
9	HBIN048372	Wogonin	Bulbus Lilii	0.042346166	0.311731844	14
10	HBIN021356	Coniferin	Radix Rehmanniae	0.054665353	0.331747919	14
11	HBIN047579	Uridine	Radix Rehmanniae	0.037946356	0.321799308	13
12	HBIN030053	Imperatorin	Bulbus Lilii	0.013973217	0.334131737	11
13	HBIN030819	Isoimperatorin	Bulbus Lilii	0.013973217	0.334131737	11
14	HBIN043533	Scutellarin	Bulbus Lilii	0.019713848	0.302931596	10
15	HBIN019475	Campesterol	Radix Rehmanniae	0.017663128	0.335740072	9
16	HBIN030377	Isoacteoside	Radix Rehmanniae	0.015708146	0.254329991	6
17	HBIN008424	3-demethylcolchicine	Bulbus Lilii	0.017251193	0.285568066	5
18	HBIN019909	Catalpol	Radix Rehmanniae	0.008063496	0.324796275	5
19	HBIN022771	Daucosterol	Radix Rehmanniae	0.007308262	0.330960854	4
20	HBIN027103	Gamma-aminobutyric acid	Radix Rehmanniae	0.021428019	0.253405995	4
21	HBIN028520	Guanosine	Radix Rehmanniae	0.014836055	0.253405995	4
22	HBIN024794	Echinacoside	Radix Rehmanniae	0.008767432	0.317406143	3
23	HBIN019775	Carthamidin	Bulbus Lilii	3.41E-04	0.272727273	2
24	HBIN022899	Decanal	Bulbus Lilii	7.43E-04	0.3089701	2
25	HBIN036819	Neryl acetate	Bulbus Lilii	0.007168459	0.272727273	2
26	HBIN042012	Rehmaglutin A	Radix Rehmanniae	0.007168459	0.252488688	2
27	HBIN042854	Salidroside	Radix Rehmanniae	8.70E-04	0.259534884	2

### 3.4. PPI network construction and module screening

**Figure 4.**
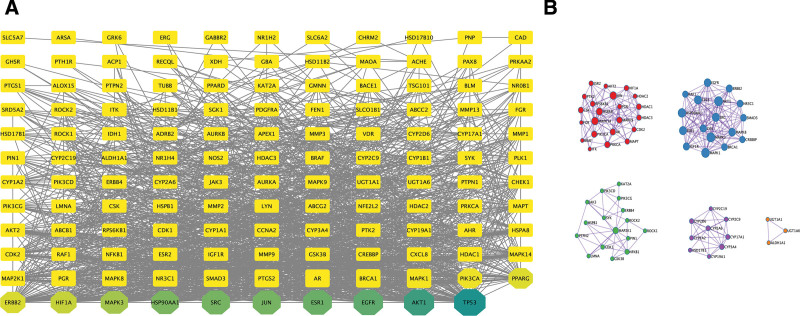
(A) BHDH Decoction and MS-related targets PPI network, (B) key modules in PPI network. MS = menopausal syndrome, PPI = protein-protein interaction.

Based on these results from drug-disease targets from STRING, we constructed a PPI network with 133 nodes and 1611 edges using Cytoscape. The results are shown in Figure 4A. (number of nodes: 133; the number of edges: 1611; average node degree: 25.5; avg. local clustering coefficient: 0.589; expected number of edges: 610; PPI enrichment *P* value: < 1.0e-16)

We used Cytoscape network analysis tool to analyze the network and adjust the size of each target in the PPI network according to its degree. The edge color is based on the total score between targets; the higher the total score, the darker the color. After obtaining the PPI network, we used the MCODE plug-in to analyze the interactions through the molecular complex detection algorithm and obtained 5 modules. The results are shown in Figure 4B. The functions of the 3 biological processes with the highest scores in the module were described according to the *P* value (Table 2). Tumor protein P53 (TP53), serine/threonine-protein kinase AKT (AKT1), epidermal growth factor receptor (EGFR), estrogen receptor 1 (ESR1), jun proto-oncogene (JUN), sarcoma gene (SRC), and HSP90AA1 in the PPI network had higher values, suggesting that they may be central targets of the BHDH Decoction in the treatment of MS.

**Table 2:** Top3 Modules function description.

GO	Function description	Log10 (P)
HSA05200	Pathways in cancer	−46.8
HSA01522	Endocrine resistance	−37.3
GO:0006468	Protein phosphorylation	−37

GO = gene ontology.

### 3.5. GO enrichment analysis and KEGG pathway

**Figure 5.**
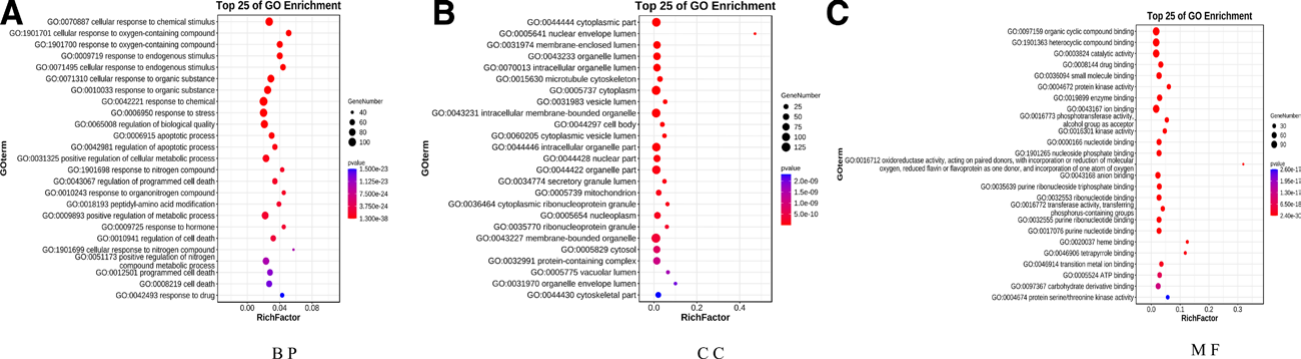
Results of GO enrichment analysis. (A) BP: biological process, (B) CC: cellular component, and (C) MF: Molecular function. GO = gene ontology.

We used the OmicShare tools to perform GO enrichment analysis on common targets of the BHDH Decoction and MS. The interaction targets were related to 5336 biological processes (BP), 499 cellular components (CC), and 730 molecular functions. We saved the first 25 results of each project in a bubble plot for further analysis (Fig. 5). It can be seen that BP is mainly enriched in cellular response to chemical stimulus, cellular response to oxygen-containing compound, response to oxygen-containing compound, response to endogenous stimulus, cellular response to endogenous stimulus, cellular response to an organic substance, response to an organic substance, response to chemicals, response to stress, regulation of biological quality, etc; CC is mainly enriched in the cytoplasmic part, nuclear envelope lumen, membrane-enclosed lumen, organelle lumen, intracellular organelle lumen, microtubule cytoskeleton, cytoplasm, vesicle lumen, intracellular membrane-bounded organelle, cell body, etc; molecular functions is mainly enriched in organic cyclic compound binding, heterocyclic compound binding, catalytic activity, drug binding, negligible molecule binding, protein kinase activity, enzyme binding, ion binding, phosphotransferase activity, alcohol group as acceptor, kinase activity, etc.

**Figure 6.**
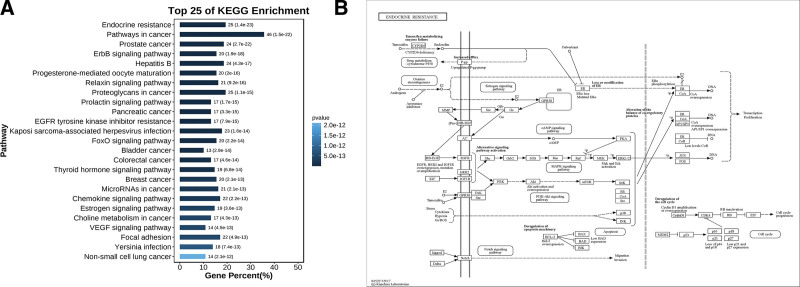
(A) Results of KEGG pathway enrichment analysis and (B) endocrine resistance pathway. KEGG = Kyoto encyclopedia of genes and genomes.

**Figure 7.**
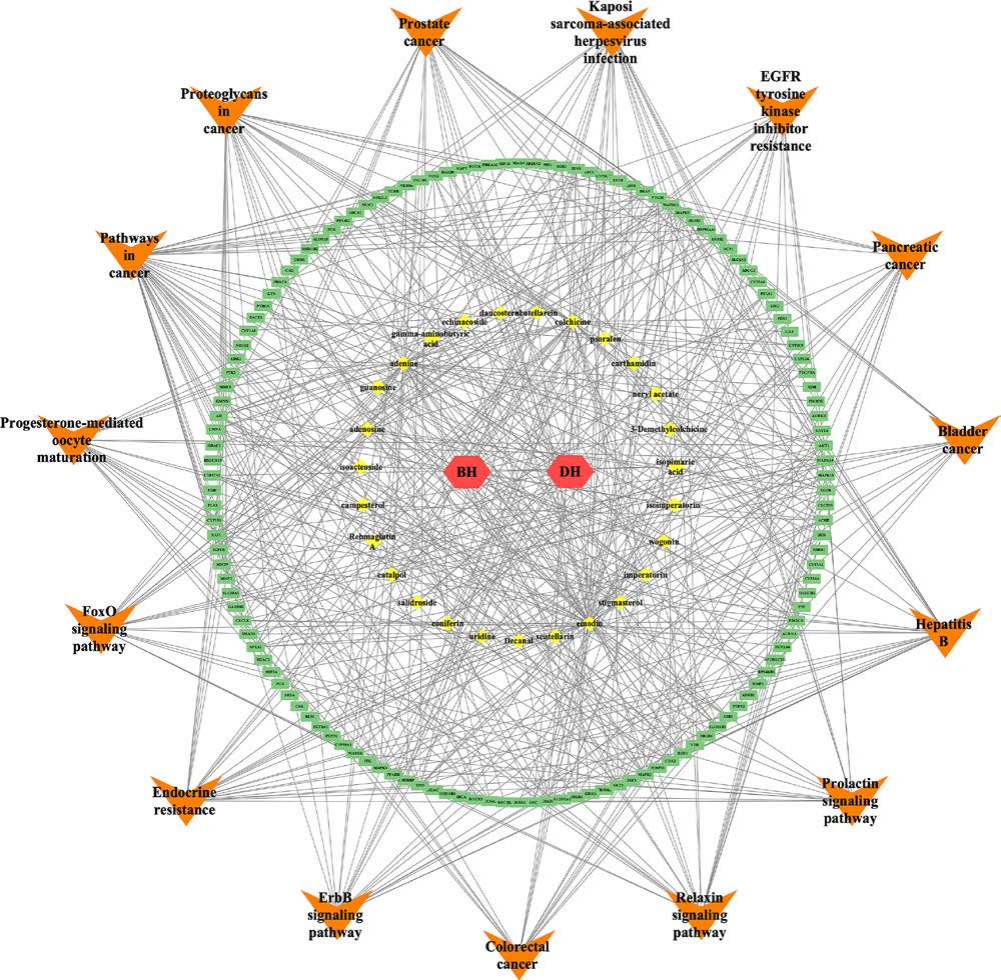
BHDH decoction - components - targets - pathways network diagram.

A total of 238 pathways were obtained by KEGG enrichment analysis (*P *< .01), and the top 25 pathways from small to large according to *P* values are shown in Figure 6 A, including endocrine resistance, pathways in cancer, prostate cancer, ErbB signaling pathway, Hepatitis B, Progesterone-mediated oocyte maturation, relaxin signaling pathway, proteoglycans in cancer, prolactin signaling pathway, and pancreatic cancer. The BHDH Decoction may act on multiple targets of multiple signaling pathways to play a role in the treatment of MS. The Endocrine resistance pathway is the most important, as shown in Figure 6B. We then used Cytoscape to construct the BHDH decoction-component-target-pathway network diagram, as shown in Figure 7.

### 3.6. Molecular docking

**Figure 8.**
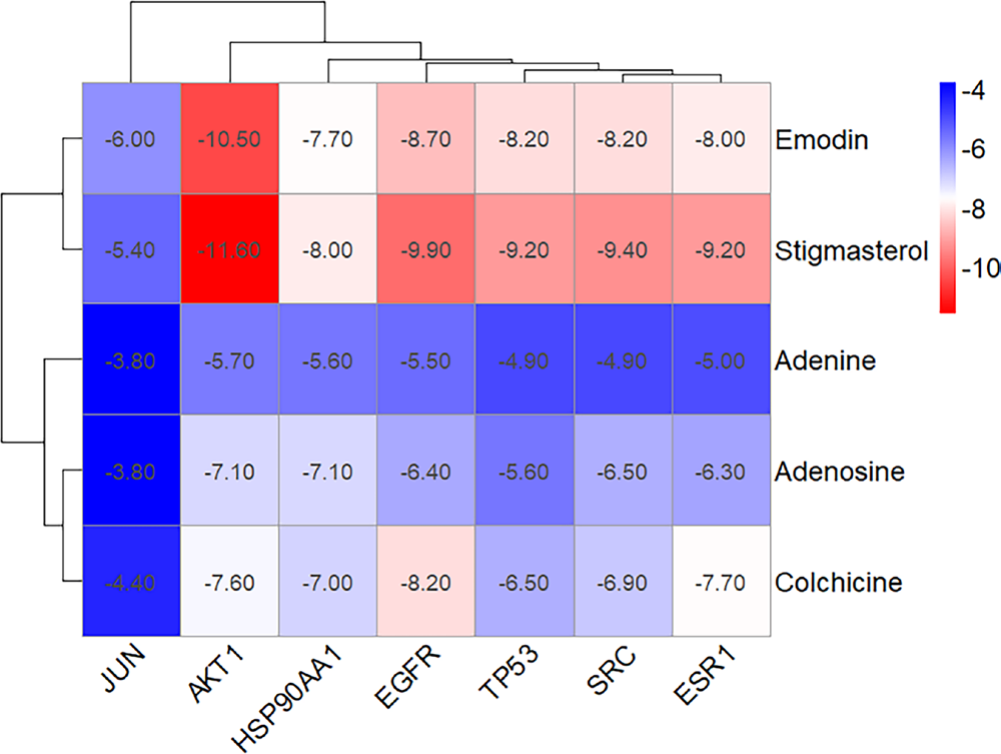
Results of molecular docking between the core components and key targets of BHDH Decoction in the treatment of MS. MS = menopausal syndrome.

**Figure 9.**
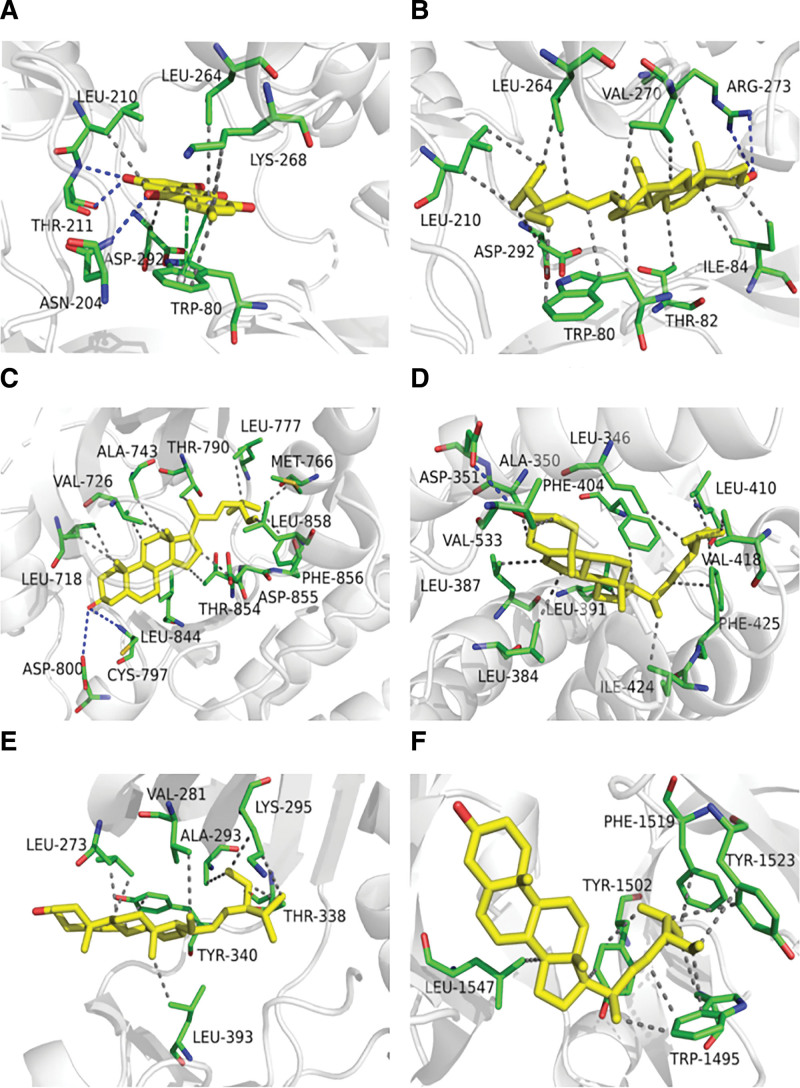
Molecular docking 3D diagram. (A) Emodin with AKT1 (binding energy = −10.5 kcal/mol). (B) Stigmasterol with AKT1 (binding energy = −11.6 kcal/mol). (C) Stigmasterol with EGFR (binding energy = −9.9 kcal/mol). (D) Stigmasterol with ESR1 (binding energy = −9.2 kcal/mol). (E) Stigmasterol with SRC (binding energy = −9.4 kcal/mol). (F) Stigmasterol with TP53 (binding energy = −9.2 kcal/mol). AKT1 = serine/threonine-protein kinase AKT, EGFR = epidermal growth factor receptor, ESR1 = estrogen receptor 1, SRC = sarcoma gene, TP53 = tumor protein P53.

The molecular ligands of the 5 main active components screened were docked with 7 key targets, and the docking parameters were as follows (Table 3). It is generally believed that when the binding energy is less than zero, compounds and proteins can spontaneously combine, and the lower the binding energy, the greater the possibility of action.^[10]^ The docking score ranges from −5.0 kcal/mol to −4.25 kcal/mol, indicating that the compound has a specific binding ability to the macromolecular target; the docking score of less than −5.0 kcal/mol and > −7.0 kcal/mol indicates that the compound has an excellent binding ability between the small molecule and the target; and the docking score less than −7.0 kcal/mol indicates a strong binding ability between the target and the compound. According to the results of molecular docking, there were 3 (8.57%) with a score between −4.25 kcal/mol and 5.0 kcal/mol;12 (34.28%) with a score of −5.0 kcal/mol between 7.0 kcal/mol; and 18 (51.43%) lower than −7.0 kcal/mol. The results of molecular docking between the core components and key targets of the BHDH Decoction in the treatment of MS are shown in Figure 8. In this study, the predicted core components and critical targets have solid binding abilities, among which emodin with AKT1, stigmasterol with AKT1, EGFR, ESR1, SRC, and TP53 have the strong binding ability, and the docking model diagram is shown in Figure 9.

**Table 3:** Docking parameters of 5 main active components.

Protein name	PDB ID	Center x	Center y	Center Z	Size x	Size y	Size z
TP53	6upt	34.39	19.02	108.746	20	20	20
AKT1	7nh5	11.96	−16.04	−13.97	20	20	20
EGFR	3poz	17.42	32.79	11.71	20	20	20
ESR1	7rs7	−4.35	8.69	19.28	20	20	20
JUN	5t01	−29.93	13.98	11.37	20	20	20
SRC	4mxo	5.57	−5.85	29.67	20	20	20
HSP90AA1	3ft5	31.89	9.91	24.91	20	20	20

AKT1 = serine/threonine-protein kinase AKT, EGFR = epidermal growth factor receptor, ESR1 = estrogen receptor 1, JUN = jun proto-oncogene, SRC = sarcoma gene, TP53 = tumor protein P53.

## 4. Discussion

Menopause is a natural physiological process in women.^[11]^ In some instances, the decline in estrogen levels caused by ovarian failure leads to a series of autonomic nervous system dysfunctions, often accompanied by hot flashes, insomnia, anxiety, depression, fatigue, and other neuropsychological symptoms.^[12]^ The extensive practice has shown that traditional estrogen replacement therapy can alleviate menopausal symptoms. However, long-term use of estrogen frequently increases the risk of breast, endometrial, and other gynecological cancers.^[13]^ Therefore, there is an urgent need to find complementary and alternative therapies, such as Traditional Chinese medicine. The BHDH Decoction has pharmacological effects, such as improving insomnia, anxiety, and depression.^[14]^ It is a representative prescription for treating MS clinically, but its molecular mechanism for treating MS has yet to be fully clarified. At the same time, BHDH Decoction is a commonly used prescription for the treatment of Lily disease with heart-lung yin deficiency and internal heat syndrome. Modern medicine also includes relevant research. Lily Bulbs have an excellent antioxidant capacity.^[15,16]^ According to Kan et al^[17]^, Lily Bulbs ameliorated oxidative stress and lipid metabolism. It can improve liver steatosis in mice fed a high-fat diet. Rehmannia inhibits adipocyte differentiation and adipogenesis.^[18]^ It also plays an antioxidative role in improving the bone structure of osteoporosis rats by improving muscle atrophy.^[19]^

The components of the BHDH Decoction were identified using HERB. At present, most studies take OB ≥ 30% and DL ≥ 0.18 as the criteria for screening ingredients, which is not comprehensive.^[20]^ Therefore, the targets of compounds in the BHDH Decoction that do not meet the standards of OB ≥ 30% and DL ≥ 0.18, but have been verified by experiments, are also retained in our study, and these targets were obtained from the Drug Bank and NPASS databases. Finally, 133 common targets were obtained. We then constructed a PPI network and screened the key modules, and found that genes such as TP53, AKT1, EGFR, ESR1, and JUN were essential in this respect. We then analyzed these targets according to the symptoms of MS. Symptoms of MS include vasomotor symptoms (hot flashes, night sweats); neuropsychiatric symptoms (insomnia, depression, memory disorders); musculoskeletal symptoms (osteoporosis, joint discomfort, and loss of muscle mass); genitourinary symptoms (vaginal atrophy and pain); and urinary tract infection.^[21]^ Estrogen plays a vital role in the full function of the female reproductive system as well as in the maintenance of bone metabolism and cognition.^[22]^ Menopause syndrome is closely associated with decreased estrogen secretion. Estrogen therapy alleviates menopause-related symptoms.^[23]^ ESR1 is the central mediator of estrogen, and its encoded transcription factor, estrogen receptor-α, plays a crucial role in regulating the hormone response of estrogen-sensitive tissues.^[24,25]^ ESR1 is broadly expressed in the central nervous system and peripheral tissues, including adipose tissue, skeletal muscle, liver, and immune cells. Skeletal muscle ERα plays a critical and protective role in regulating mitochondrial function, metabolic homeostasis, and insulin activity.^[26]^ ESR1 may play a role in susceptibility to the depressive mood in postmenopausal women.^[27]^ Estrogen mediates its effects by binding to ESR1, leading to the expression of genes that control cell proliferation and survival.^[28]^ ESR1 gene polymorphisms are associated with metabolic syndromes (including obesity, diabetes, dyslipidemia, and hypertension) in postmenopausal women in China.^[29]^ DHTKD1 and RBBP4 may be involved in postmenopausal osteoporosis by regulating mitochondrial dysfunction and interacting with ESR1, respectively.^[30]^ The distribution of body fat in postmenopausal women changes and subcutaneous fat is transferred to abdominal visceral fat, resulting in central obesity and metabolic syndrome. AKT1 affects cellular homeostasis and is considered to be a survival factor that suppresses apoptosis. Studies have shown that the proliferative capacity of adipose tissue in postmenopausal women is lower than that in premenopausal women.^[31–33]^ The content of AKT1 in the subcutaneous adipose tissue of postmenopausal women is lower than that in premenopausal women, which is related to the slow proliferative activity of AKT1 in subcutaneous adipose tissue obtained from the abdomen.^[33]^ The ESR1 signaling pathway in adipose tissue is likely to be followed by the activation of AKT1, leading to more proliferative or antiapoptotic processes.^[33]^ Combined with this literature, the targets of BHDH Decoction mainly focus on the endocrine resistance pathway, and we speculate that BHDH Decoction can enhance the sensitivity of ESR1, which not only reduces the side effects of hormone replacement therapy but also regulates fat metabolism and reduces systemic inflammation. MS is an endocrine disease associated with aging. TP53, as a “guardian gene,” has been shown to be a good target for preventing age-related disorders.^[34]^ The physiological function of EGFR is to regulate epithelial tissue development and homeostasis and is also a crucial regulator of autophagy.^[35]^ EGFR signaling is associated with insulin resistance and liver, muscle, and adipose inflammation. Inhibition of EGFR can improve tyrosine phosphorylation of insulin receptors and insulin receptor substrates in obese mice.^[36]^ EGFR signaling and its downstream pathways provide new therapeutic targets for regulating sleep.^[37]^ Insomnia is a common condition in menopausal women. The Jun family includes c-Jun, JunB, and JunD. Jun N-terminal kinase, a member of the MAPK family, is associated with the inhibition of cell proliferation.^[38,39]^ Increasing evidence suggests that the MAPK/ERK pathway is involved in the pathogenesis of depression and mediates the regulation of the circadian system.^[40]^ This suggests that mental symptoms such as insomnia, anxiety, and depression during menopause may be related to the MAPK/ERK pathway.

In addition, we screened modules in the PPI network and described their functions. According to the *P* value, the functions of the 3 biological processes with the highest scores in the 5 modules were pathways in cancer, endocrine resistance, and protein phosphorylation.^[41]^ The endocrine resistance signaling pathway is currently considered one of the pathways in the pathogenesis of MS. To further evaluate the complex relationships among different components, targets, and pathways of the BHDH Decoction in the treatment of MS, GO and KEGG analyses were conducted. According to the *P* value, the most important biological processes are cellular response to chemical stimuli, cellular response to oxygen-containing compounds, response to oxygen-containing compounds, response to endogenous stimuli, and cellular response to organic substances. These biological processes are closely related to MS.^[23,42]^

Furthermore, depending on the degree, BC, and CC of the BHDH decoction-components-target-pathways network, we predicted emodin, adenine, colchicine, adenosine, stigmasterol, and emodin to be the core components of the BHDH Decoction. Emodin exhibits a variety of pharmacological benefits, including anticancer, anti-inflammatory, antioxidant, antimicrobial, immunosuppressive, and osteogenesis promotion activity, suggesting it could be used for the treatment of menopause-related anxiety, depression, osteoporosis, and other symptoms.^[43]^ Osteoarthritis is a frequently occurring disease in menopausal women. Colchicine is commonly used for the treatment of gout. Colchicine can reduce cardiovascular risk owing to its anti-inflammatory effects.^[44]^ Furthermore, adenosine modulates vascular homeostasis in the heart.^[45]^ Stigmasterol is a plant sterol reported to have a variety of physiological functions.^[46]^ It can reduce inflammation by regulating the MAPK/NF-κB ROCK1 pathway. The oxidation products of stigmasterol interfere with the female sex hormone 17β-estradiol in human breast and endometrial cells.^[47]^ Stigmasterol Causes Ovarian Cancer Cell Apoptosis by Inducing Endoplasmic Reticulum and Mitochondrial Dysfunction.^[48]^ Stigmasterol is also a potential metabolic regulator of neurodegenerative diseases.^[49]^ BHDH Decoction may treat MS through possible signaling pathways, including endocrine resistance, the ErbB signaling pathway, Hepatitis B, Progesterone-mediated oocyte maturation, and the relaxin signaling pathway. The Endocrine resistance signaling pathway is the most critical, and CCL2 activates the PI3K/Akt/mTOR pathway, which is a classical endocrine resistance pathway.^[50]^

Finally, Autodock Vina 1.1.2 software for docking the central components and targets. The docking results showed that the central active components of BHDH have an excellent binding activity to the central targets of MS. In this study, the predicted core components and critical targets have strong binding abilities, among which emodin with AKT1, stigmasterol with AKT1, EGFR, ESR1, SRC, and TP53 have solid binding abilities. In summary, BHDH Decoction alleviates MS through its main components, emodin, and stigmasterol, acting on AKT1, EGFR, ESR1, SRC, TP53, and other targets, as well as endocrine resistance and other pathways. The results of molecular docking further suggest that the chemical components of the BHDH Decoction may have good prospects as therapeutic drugs for MS and also provide molecular research ideas for subsequent basic research.

## 5. Conclusion

Based on network pharmacology and molecular docking, this study explains the practical components of the BHDH Decoction and its related targets and pathways for treating MS. The central components may be emodin, adenine, colchicine, adenosine, and stigmasterol. It probably pairs core targets such as ESR1, AKT1, TP53, EGFR, and JUN to regulate endocrine resistance, ErbB signaling pathway, Hepatitis B, Progesterone-mediated oocyte maturation, and relaxin signaling pathway, which play a role in the treatment of MS. In addition, we demonstrated an excellent combination of hub components and hub targets through molecular docking, which provides a reference for exploring the pharmacological effects of DHDH Decoction. This study preliminarily reveals the mechanism of BHDH Decoction in treating Menopausal Syndrome. It provides a reference for in vitro and in vivo research and clinical application of BHDH Decoction in treating MS.

## Author contributions

**Conceptualization:** Mingmin Tian, Gaofeng Liu.

**Data curation:** Anming Yang, Guangjie Liu.

**Formal analysis:** Qinwei Lu, Xin Zhang.

**Investigation:** Mingmin Tian.

**Methodology:** Anming Yang, Xin Zhang.

**Project administration:** Mingmin Tian, Gaofeng Liu.

**Resources:** Mingmin Tian.

**Software:** Guangjie Liu, Xin Zhang.

**Supervision:** Mingmin Tian, Gaofeng Liu.

**Validation:** Qinwei Lu.

**Writing-original draft:** Mingmin Tian, Anming Yang.

**Writing-review & editing:** Guangjie Liu, Gaofeng Liu.

## Supplementary Material

## References

[1] Wang XJ. Treatment of climacteric syndrome with traditional Chinese and Western medicine. J Pract Intern Med Tradit Chin Med. 2015;29:85–7.

[2] Liu YF Wang TF. Review on the treatment of perimenopausal syndrome with integrated traditional Chinese and Western medicine. J Beijing Univ Tradit Chin Med. 2022;45:15–20.

[3] Johnson A Roberts L Elkins G. Complementary and alternative medicine for menopause. J Evid Based Integr Med. 2019;24:2515690X–19829380. 10.1177/2515690X19829380 PMC6419242 30868921

[4] Han X Zhang WY Dang ZB. Theoretical study of traditional Chinese medicine on female climacteric syndrome. Inner Mongolia Tradit Chin Med. 2014;33:130.

[5] Liu WQ Wu SY. Overview of Baihe Dihuang decoction in the treatment of psychosis. Chin J Clin Med. 2019;31:1816–9.

[6] Pan WC Pan J Xue XY. Study on the chemical constituents of modern Baihe Dihuang decoction. Shizhen National Med. 2021;32:1884–8.

[7] Zhu SH Xie M. Clinical research progress of Baihe Dihuang decoction. Chin Pharm. 2021;24:2081–8.

[8] Fang S Dong L Liu L. HERB: a high-throughput experiment- and reference-guided database of traditional Chinese medicine. Nucleic Acids Res. 2021;49:D1197–206. 33264402 10.1093/nar/gkaa1063 PMC7779036

[9] Lin D Zeng Y Tang D. Study on the mechanism of Liuwei Dihuang pills in treating Parkinson’s disease based on network pharmacology. Biomed Res Int. 2021;2021:4490081. 34746302 10.1155/2021/4490081 PMC8568527

[10] Lai SJ Wang DY Li TL. Molecular docking and network pharmacology in the treatment of microvascular angina pectoris with Hypericum perforatum. Chin J Tradit Chin Med. 2021;46:6474–83. 10.19540/j.cnki.cjcmm.20210902.401 34994140

[11] Zhou XD Zheng Y Sharma R. Total polysaccharides of lily bulb ameliorate menopause-like behavior in ovariectomized mice: multiple mechanisms distinct from estrogen therapy. Oxid Med Cell Longev. 2019;2019:6869350. 31428228 10.1155/2019/6869350 PMC6683782

[12] Kalmbach DA Cheng P Arnedt JT. Improving daytime functioning, work performance, and quality of life in postmenopausal women with insomnia: comparing cognitive behavioral therapy for insomnia, sleep restriction therapy, and sleep hygiene education. J Clin Sleep Med. 2019;15:999–1010. 31383238 10.5664/jcsm.7882 PMC6622507

[13] Wang Y Sun J Zhang K. Black tea and D. candidum extracts play estrogenic activity via estrogen receptor α-dependent signaling pathway. Am J Transl Res. 2018;10:114–25. 29422998 PMC5801351

[14] Zhu SH Xie M. Research progress on pharmacological effects of Baihe Dihuang Decoction. J Guangzhou Univ Tradit Chin Med. 2022;39:719–26.

[15] Tang YC Liu YJ He GR. Comprehensive analysis of secondary metabolites in the extracts from different lily bulbs and their antioxidant ability. Antioxidants (Basel). 2021;10:1634. 34679768 10.3390/antiox10101634 PMC8533310

[16] Liang ZX Zhang JZ Xin C. Analysis of edible characteristics, antioxidant capacities, and phenolic pigment monomers in Lilium bulbs native to China. Food Res Int. 2022;151:110854. 34980390 10.1016/j.foodres.2021.110854

[17] Kan J Hui Y Xie W. Lily bulbs’ polyphenols extract ameliorates oxidative stress and lipid accumulation in vitro and in vivo. J Sci Food Agric. 2021;101:5038–48. 33570774 10.1002/jsfa.11148

[18] Jiang L Zhang NX Mo W. Rehmannia inhibits adipocyte differentiation and adipogenesis. Biochem Biophys Res Commun. 2008;371:185–90. 18395006 10.1016/j.bbrc.2008.03.129

[19] Ou L Kang W Zhang J. Effects of Rehmannia glutinosa polysaccharides on bone tissue structure and skeletal muscle atrophy in rats with disuse. Acta Cir Bras. 2021;36:e360403. 34008744 10.1590/ACB360403 PMC8128353

[20] Ren Y Deng YJ Ma HB. Research progress and challenges of network pharmacology in the field of traditional Chinese medicine. Chin Tradit Herbal Drugs. 2020;51:4789–97.

[21] Chinnappan SM George A Evans M. Efficacy of Labisia pumila and Eurycoma longifolia standardised extracts on hot flushes, quality of life, hormone and lipid profile of peri-menopausal and menopausal women: a randomised, placebo-controlled study. Food Nutr Res. 2020;64:3665. 10.29219/fnr.v64.3665 PMC7534949 33061884

[22] Tecalco-Cruz AC Zepeda-Cervantes J Ortega-Domínguez B. Estrogenic hormones receptors in Alzheimer’s disease. Mol Biol Rep. 2021;48:7517–26. 34657250 10.1007/s11033-021-06792-1

[23] Dietz BM Hajirahimkhan A Dunlap TL. Botanicals and their bioactive phytochemicals for women’s health. Pharmacol Rev. 2016;68:1026–73. 27677719 10.1124/pr.115.010843 PMC5050441

[24] Liu X Huang J Lin H. ESR1 PvuII (rs2234693 T>C) polymorphism and cancer susceptibility: evidence from 80 studies. J Cancer. 2018;9:2963–72. 30123365 10.7150/jca.25638 PMC6096366

[25] Plassais J Kim J Davis BW. Whole genome sequencing of canids reveals genomic regions under selection and variants influencing morphology. Nat Commun. 2019;10:1489. 30940804 10.1038/s41467-019-09373-w PMC6445083

[26] Hevener AL Ribas V Moore TM. The impact of skeletal muscle ERα on mitochondrial function and metabolic health. Endocrinology. 2020;161:bqz017. 32053721 10.1210/endocr/bqz017 PMC7017798

[27] Różycka A Słopień R Słopień A. The MAOA, COMT, MTHFR and ESR1 gene polymorphisms are associated with the risk of depression in menopausal women. Maturitas. 2016;84:42–54. 26620113 10.1016/j.maturitas.2015.10.011

[28] Martin LA Ribas R Simigdala N. Discovery of naturally occurring ESR1 mutations in breast cancer cell lines modelling endocrine resistance. Nat Commun. 2017;8:1865. 29192207 10.1038/s41467-017-01864-y PMC5709387

[29] Zhao L Fan X Zuo L. Estrogen receptor 1 gene polymorphisms are associated with metabolic syndrome in postmenopausal women in China. BMC Endocr Disord. 2018;18:65. 30217154 10.1186/s12902-018-0289-4 PMC6137943

[30] Yang C Ren J Li B. Identification of gene biomarkers in patients with postmenopausal osteoporosis. Mol Med Rep. 2019;19:1065–73. 30569177 10.3892/mmr.2018.9752 PMC6323213

[31] Liu F Li L Li Y. Overexpression of SENP1 reduces the stemness capacity of osteosarcoma stem cells and increases their sensitivity to HSVtk/GCV. Int J Oncol. 2018;53:2010–20. 30226577 10.3892/ijo.2018.4537 PMC6192779

[32] Wu Y Lee MJ Ido Y. High-fat diet-induced obesity regulates MMP3 to modulate depot- and sex-dependent adipose expansion in C57BL/6J mice. Am J Physiol Endocrinol Metab. 2017;312:E58–71. 27879248 10.1152/ajpendo.00128.2016 PMC5283879

[33] Kangas R Morsiani C Pizza G. Menopause and adipose tissue: miR-19a-3p is sensitive to hormonal replacement. Oncotarget. 2017;9:2279–94. 29416771 10.18632/oncotarget.23406 PMC5788639

[34] Pawge G Khatik GL. p53 regulated senescence mechanism and role of its modulators in age-related disorders. Biochem Pharmacol. 2021;190:114651. 34118220 10.1016/j.bcp.2021.114651

[35] Sigismund S Avanzato D Lanzetti L. Emerging functions of the EGFR in cancer. Mol Oncol. 2018;12:3–20. 29124875 10.1002/1878-0261.12155 PMC5748484

[36] Ardestani A Li S Annamalai K. Neratinib protects pancreatic beta cells in diabetes. Nat Commun. 2019;10:5015. 31676778 10.1038/s41467-019-12880-5 PMC6825211

[37] Lee DA Liu J Hong Y. Evolutionarily conserved regulation of sleep by epidermal growth factor receptor signaling. Sci Adv. 2019;5:eaax4249. 31763451 10.1126/sciadv.aax4249 PMC6853770

[38] Mohammad H Marchisella F Ortega-Martinez S. JNK1 controls adult hippocampal neurogenesis and imposes cell-autonomous control of anxiety behaviour from the neurogenic niche. Mol Psychiatry. 2018;23:362–74. 27843149 10.1038/mp.2016.203 PMC5794884

[39] Xie P Horio F Fujii I. A novel polysaccharide derived from algae extract inhibits cancer progression via JNK, not via the p38 MAPK signaling pathway. Int J Oncol. 2018;52:1380–90. 29512724 10.3892/ijo.2018.4297 PMC5873927

[40] Wang XL Yuan K Zhang W. Regulation of circadian genes by the MAPK pathway: implications for rapid antidepressant action. Neurosci Bull. 2020;36:66–76. 30859414 10.1007/s12264-019-00358-9 PMC6940409

[41] Vella D Marini S Vitali F. MTGO: PPI network analysis via topological and functional module identification. Sci Rep. 2018;8:5499. 29615773 10.1038/s41598-018-23672-0 PMC5882952

[42] Lee E Jang M Lim TG. Selective activation of the estrogen receptor-β by the polysaccharide from Cynanchum wilfordii alleviates menopausal syndrome in ovariectomized mice. Int J Biol Macromol. 2020;165(Pt A):1029–37. 32991896 10.1016/j.ijbiomac.2020.09.165

[43] Cui Y Chen LJ Huang T. The pharmacology, toxicology and therapeutic potential of anthraquinone derivative emodin. Chin J Nat Med. 2020;18:425–35. 32503734 10.1016/S1875-5364(20)30050-9

[44] Robinson PC Terkeltaub R Pillinger MH. Consensus statement regarding the efficacy and safety of long-term low-dose colchicine in gout and cardiovascular disease. Am J Med. 2022;135:32–8. 34416165 10.1016/j.amjmed.2021.07.025 PMC8688259

[45] Soattin L Lubberding AF Bentzen BH. Inhibition of adenosine pathway alters atrial electrophysiology and prevents atrial fibrillation. Front Physiol. 2020;11:493. 32595514 10.3389/fphys.2020.00493 PMC7304385

[46] Miras-Moreno B Sabater-Jara AB Pedreño MA. Bioactivity of phytosterols and their production in plant in vitro cultures. J Agric Food Chem. 2016;64:7049–58. 27615454 10.1021/acs.jafc.6b02345

[47] Nascimento EBM Konings M Schaart G. In vitro effects of sitosterol and sitostanol on mitochondrial respiration in human brown adipocytes, myotubes and hepatocytes. Eur J Nutr. 2020;59:2039–45. 31317217 10.1007/s00394-019-02052-y PMC7351807

[48] Bae H Song G Lim W. Stigmasterol causes ovarian cancer cell apoptosis by inducing endoplasmic reticulum and mitochondrial dysfunction. Pharmaceutics. 2020;12:488. 32481565 10.3390/pharmaceutics12060488 PMC7356731

[49] Sharma N Tan MA An SSA. Phytosterols: potential metabolic modulators in neurodegenerative diseases. Int J Mol Sci. 2021;22:12255. 34830148 10.3390/ijms222212255 PMC8618769

[50] Li D Ji H Niu X. Tumor-associated macrophages secrete CC-chemokine ligand 2 and induce tamoxifen resistance by activating PI3K/Akt/mTOR in breast cancer. Cancer Sci. 2020;111:47–58. 31710162 10.1111/cas.14230 PMC6942430

